# Nature-Inspired Solutions for Sustainable Mining: Applications of NIAs, Swarm Robotics, and Other Biomimicry-Based Technologies

**DOI:** 10.3390/biomimetics10030181

**Published:** 2025-03-14

**Authors:** Joven Tan, Noune Melkoumian, David Harvey, Rini Akmeliawati

**Affiliations:** 1 Discipline of Mining and Petroleum Engineering, School of Chemical Engineering, The University of Adelaide, Adelaide 5005, Australia; noune.melkoumian@adelaide.edu.au; 2 School of Electrical and Mechanical Engineering, The University of Adelaide, Adelaide 5005, Australia; david.harvey@adelaide.edu.au (D.H.); rini.akmeliawati@adelaide.edu.au (R.A.)

**Keywords:** biomimicry, nature-inspired algorithms, swarm robotics, mining automation, collective intelligence, sustainable mining

## Abstract

Environmental challenges, high safety risks and operational inefficiencies are some of the issues facing the mining sector. The paper offers an integrated viewpoint to address these issues by combining swarm robotics, nature-inspired algorithms (NIAs) and other biomimicry-based technologies into a single framework. It presents a systematic classification of each methodology, emphasizing their key advantages and disadvantages as well as considering real-life mining application scenarios, including hazard detection, autonomous transportation and energy-efficient drilling. Case studies are citied to demonstrate how these methodologies work together, and an extensive comparison table considering their applications at mines, such as Boliden, Diavik Diamond Mine, Olympic Dam and others, presents a summary of their scalability and practicality. This paper highlights future directions such as multi-robot coordination and hybrid NIAs, to improve operational resilience and sustainability. It also provides a broad overview of biomimicry and critically examines unresolved issues like real-time adaptation, parameter tuning and mechanical wear. The paper aims to offer a comprehensive insight into using bio-inspired models to enhance mining efficiency, safety and environmental management, while proposing a road map for resolving the issues that continue to be a hurdle for wide adaptation of these technologies in the mining industry.

## 1. Introduction

The mining sector as the provider of vital raw materials for various global industries, such as building, manufacturing and energy production, is a key component of global industrial activities [1,2]. However, due to its nature and with the discovery and extraction of new deposits, the mining sector continues to face new challenges such as managing increasingly complex deposits that are in deeper or remote geological environments [3,4,5], adhering to stricter environmental [6,7,8] and occupational health and safety regulations [9,10], etc. Current traditional automated techniques are more sophisticated than the earlier methods [11,12], but they often fall short of meeting operational flexibility and sustainability requirements of contemporary stakeholders. To achieve better effectiveness and resilience, researchers are increasingly looking for solutions inspired by nature. Nature-inspired designs are essentially about using biological strategies, which have developed and undergone optimization for millions of years and have withstood the test of time, to solve engineering problems [13]. In the mining sector, this can involve addressing difficult working conditions and intricate geological uncertainties, and developing engineering solutions using bio-inspired systems could be a highly effective approach.

To address major issues facing contemporary mining, such as issues on safety, environmental impact, cost-effectiveness, energy consumption and real-time adaptability, this article considers three main categories of nature-inspired methodologies: biomimicry-based technologies, NIAs and swarm robotics. Biomimicry uses functional and structural ideas from biological evolution to create new tools, materials or procedures [14]. By transferring naturally optimized adaptations, such as efficient burrowing, sensory acuity, or self-assembling structures into mining technologies, biomimetic approaches can reduce energy consumption, mitigate environmental impact, and increase resilience in challenging geological settings. Nature-inspired algorithms (NIAs) are a class of algorithms that mimic biological, evolutionary or social optimization heuristics for applications to logistics planning, resource scheduling and exploration, to name a few [15,16]. Swarm robotics applies models and ideas from animal swarm intelligence to distributed multi-robot systems offering resilience and flexibility, which is important for settings where there are no reliable communication systems [17].

These three methodologies often are being considered individually, especially in studies that focus on their theoretical underpinnings and related case studies. For example, in studies on the effectiveness of NIAs for mine planning under uncertainty [18,19,20], on applying biomimicry robots to significantly reduce excavation energy in soft soils [21,22], or on investigating how swarm robotics might be used to automate transportation [23,24,25]. Seldom have these strategies been thoroughly combined to show their complementary qualities and joint capability to transform mining operations [26]. Despite significant advances in automation and artificial intelligence applications in the mining industry, such as deployment of drones, autonomous haul trucks, drilling robots and underground inspection systems, these efforts have largely remained isolated. Prior research has typically focused on remote communications between individual robotic platforms or fleets, without considering the potential benefits of combining NIAs, biomimicry-based technologies, and swarm robotics into a cohesive framework.

In this paper, we present a thorough examination of nature-inspired algorithms (NIAs), swarm robotics and other biomimicry-based technologies, emphasizing their potential to tackle present and future mining industry challenges. After outlining the fundamental ideas and common uses of NIAs, Section 2 focuses on how they can be utilized to optimize mining operations. After discussing the theoretical underpinnings and applications of swarm robotics, Section 3 shows how it can be applied to both open-pit and underground mining. Section 4 looks at the key biomimicry-based innovations and how they relate to mining operations such as danger identification and tunnelling. The mining industry faces challenges like energy consumption, real-time adaptability, and environmental concerns. Section 5 bridges these three paradigms and provides cooperative solutions. Section 6 concludes with findings and suggestions for further study.

## 2. Nature-Inspired Algorithms in Mining

### 2.1. Overview of NIA Principles

**Figure 1:**
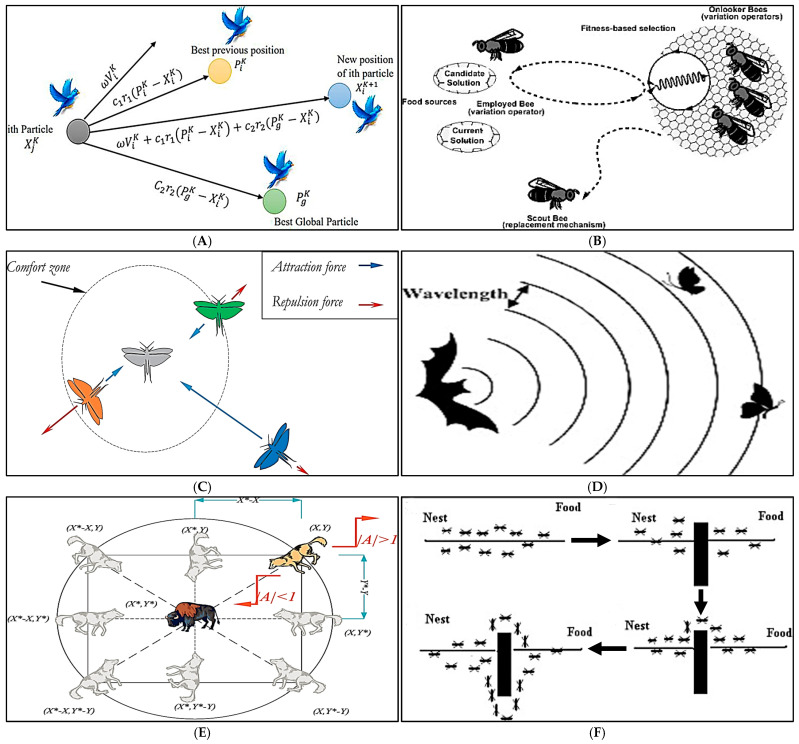

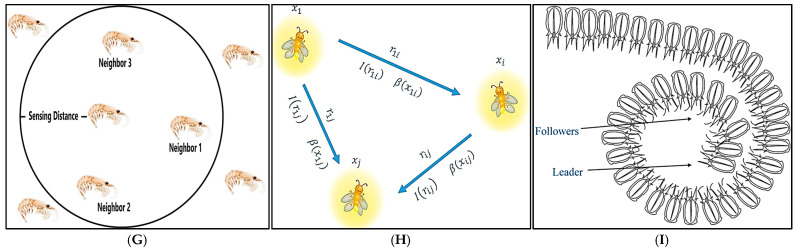
Bio-inspired mechanisms of NIAs. (Image (**A**–**I**) courtesy of Elsevier publisher) (**A**) Particle Swarm Optimization (PSO) mimics bird flocking and fish schooling [28]; (**B**) Artificial Bee Colony (ABC) models bee foraging behavior [29]; (**C**) Grasshopper Optimization Algorithm (GOA) simulates swarm dynamics of grasshoppers [30]; (**D**) Bat Algorithm (BA) uses bat echolocation for multi-dimensional optimization [31]; (**E**) Grey Wolf Optimization (GWO) emulates hunting and social hierarchy of grey wolves [32]; (**F**) Ant Colony Optimization (ACO) arrows replicate the foraging behavior of ants, i.e., moving from the nest to the food, over obstacles and back to the nest [33]; (**G**) Krill Herd Algorithm (KHA) mimics herding behavior of krill [34]; (**H**) Firefly Algorithm (FA) models firefly flashing patterns for optimization [35]; and (**I**) Salp Swarm Algorithm (SSA) captures leader–follower dynamics of salp chains [36]. (Note: *x* (position), *v* (velocity), ω (inertia weight), $c_{1}c_{2}$ (cognitive and social coefficients), $r_{1}r_{2}$ (random numbers in [0, 1]) in PSO; hive dynamics in ABC; attraction and repulsion zones in GOA; wavelength and pulse rate in BA; hierarchy levels (alpha, beta, delta, and omega) in GWO; paths *A* and *B* in ACO; interaction points 1 to 6 in KHA; $\beta_{0}$ (brightness) and $I_{0}$ (intensity) in FA; and leader–follower positions in SSA).

NIAs provide heuristic solutions to difficult optimization problems by employing tactics inspired by biological models and processes such as avian socialization, insect foraging or evolutionary adaptation [27]. This study examines the performance efficiency of NIAs in multi-objective and data-intensive mining settings. A thorough introduction to the equations and pseudocodes underlying these techniques is presented in the paper by Tan et al. [20]. It has been shown that a number of important aspects in mining, such as exploration, planning, logistics, safety and environmental sustainability, can be addressed by NIAs [20]. This section examines recent NIA applications that have shown measurable advantages in lowering risk, raising profitability and increasing productivity across the mining industry. Various applications of NIAs in the mining sector, highlighting their innovative attributes, are illustrated in Figure 1.

Even though NIAs have proven to be flexible in a variety of industrial and service applications, mining has its share of difficulties, including the requirement for reliable scheduling in the face of uncertainty and large-scale block models with shifting geological conditions. Using these algorithms in mining environments has several advantages, including better safety procedures, lower operating costs, and more effective resource allocation. The specific applications of NIAs for fundamental mining tasks such as production planning, route scheduling, and real-time danger identification is covered in the next section.

### 2.2. Core NIAs in Mining Contexts

Although the earlier study [20] presents a thorough summary on the number of NIAs, it does not include in-depth comparisons of newer algorithms or discussions on their recent field applications. Section 2.2 addresses this gap by examining each NIA’s advantages, mining applications, and associated limitations. Table 1 underscores key mining-specific complexities and provides fresh insights into why certain NIAs might fail at the pilot scale, thereby highlighting the importance of robust, adaptable solutions suited to dynamic operational conditions.

Although abrupt geological changes and communication breakdowns may limit the effectiveness of NIAs, they generally have a lot of potential for mining optimization. Technologies like swarm robots may be able to assist in resolving these problems. The fundamentals of swarm robotics will be covered in the next section along with examples on how they can be applied in conjunction with NIAs in the harsh and remote settings that are common in many mining operations.

### 2.3. Critical Limitations and Failure Modes of NIAs

Despite multi-objective successes, most NIAs are not easily adaptable to real-time conditions, and scalability remains an open challenge. Unlike the earlier study [20], current investigation highlights also the additional practical mining-specific constraints that these algorithms face, such as hazard regulation and operator training costs. Scalability is a significant challenge, since complex route networks and large block models can occasionally consume excessive amounts of processing power and hybrid heuristics, and parallelization is still underutilized. Moreover, many NIAs rely heavily on carefully calibrated parameters, such as inertia weights in PSO [37,38,39,40] or pheromone evaporation rates in ACO [45,46,47,48]. These parameters may lead to early convergence or less-than-ideal solutions when conditions change dynamically. Traditional NIAs also find it difficult to quickly adapt to abrupt geological or operational changes, because most algorithms were first developed for offline applications [41,42,49,50]. Furthermore, simultaneously managing discrete variables such as block ordering, and continuous factors such as haulage speed, introduces complexity and frequently necessitates domain-specific modifications. Moreover, NIAs are often limited in their applicability in the mining industry because of failure to consider external constraints, such as safety regulations, environmental concerns and community impacts.

Together, these drawbacks limit NIAs’ ability to sustain reliable performance when confronted with abrupt geological shifts, heavy data loads, and strict real-time constraints in mining. NIAs’ other critical challenges include finely tuned parameters, scalability issues, and difficulty adjusting in real time; consequently, they are less effective as standalone tools in complex mining scenarios. Therefore, in rapidly evolving conditions, using NIAs by themselves may not suffice to ensure fully flexible and dependable mining solutions. This limitation suggests that hybrid or adaptive mechanisms are needed to adjust parameters intelligently as mining challenges change. Section 5 presents a detailed discussion on how these integrated solutions could improve the resilience and performance of NIAs in the mining industry.

## 3. Swarm Robotics and Automation

### 3.1. Overview of Swarm Robotics Principles

**Figure 2:**
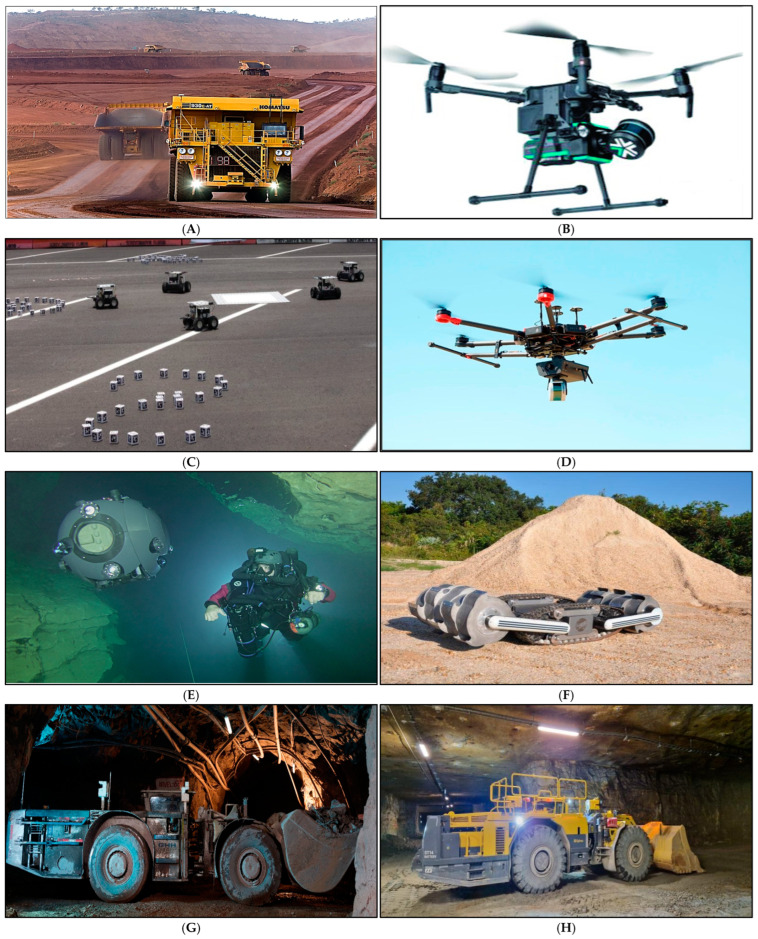
Examples of advanced robotic technologies relevant to single- or multi-robot (swarm) mining applications: (**A**) Komatsu’s FrontRunner Autonomous Haulage System (AHS) for surface mining [55] (image courtesy of Komatsu Mining Corporation); (**B**) Exyns A3R, a LiDAR- and SLAM-equipped drone for underground mapping [56] (image courtesy of Canadian Science publisher, CC0); (**C**) NASA’s “Swarmies”, illustrating collaborative resource collection [57] (image courtesy of NASA, CC0); (**D**) CSIRO Hovermaps on a DJI M600 UAV platform for subterranean exploration [58] (image courtesy of Elsevier publisher, CC0); (**E**) the UX-1Neo underwater robot from the UNEXUP project for flooded mines [59] (image courtesy of the UNEXUP project); (**F**) RASSOR, a modular robot designed for extraterrestrial regolith excavation [60] (image courtesy of Elsevier publisher); (**G**) Sandvik’s AutoMine System for autonomous loaders and trucks in underground operations [61] (image courtesy of MDPI publisher, CC0); and (**H**) NEXGEN SIMS robots for autonomous drilling, hauling, and inspection [62]. (Note: While (**C**) is explicitly developed as a multi-robot swarm platform, the others (**A**,**B**,**D**–**H**) typically operate as single units or under centralized control but have been proposed or designed with the potential to be scaled into decentralized swarm frameworks, thus collectively highlighting how current mining robotics may evolve toward collaborative, swarm-based solutions).

Large numbers of relatively simple robots that share decision-making abilities through decentralized mechanisms are the subject of swarm robotics, which is often inspired by the behaviors of social insects, birds, schools of fish, etc. [53]. Although previous study [20] summarizes basic concepts of decision-making, navigation, spatial organization and formation control, it provides only a basic understanding on how these techniques might be used in large-scale or high-risk industries like mining. Section 3.1 goes beyond this by focusing on the development and application of swarm robotics in modern mining environments, where communication failures, complex tunnel networks and changing geological conditions present unique challenges [54]. The following section explores applications of various advanced robotic systems in the mining sector, highlighting their innovative features, which are illustrated in Figure 2.

Notwithstanding the difficulties associated with mining, particularly with underground mining due to dusty tunnels, unstable rock formations, poor vision, and occasionally inconsistent communications, these applications demonstrate that the versatility of swarm intelligence can be beneficial. Swarm robotics is the ideal solution despite these difficulties because tiny robots can navigate in confined spaces, reducing the possibility of people being exposed to hazardous situations, and they have decentralized autonomy that keeps working even in the event of certain communications failures.

### 3.2. Core Swarm Robotics in Mining Contexts

Building upon the potential for scaling autonomous platforms (as depicted in Figure 2) from stand-alone solutions to a decentralized swarm architecture, this section examines the current state and emerging developments of swarm robotics in mining. By leveraging collective intelligence principles, multiple robotic units can coordinate haul routes, exchange sensor data, and swiftly adapt to shifting geological conditions. Such collaborative capabilities have the potential to enhance operational efficiency and strengthen safety standards within mining environments; these are achievements that often exceed those feasible with isolated systems.

Although swarm robotics has seen extensive adoption across a range of industries, such as agriculture (SAGA project) [63], construction (Fiberbots) [64] and KALI [65], transportation, and medicine (millirobots) [66], its implementation in the mining sector is relatively recent. Nevertheless, early applications have already generated noteworthy improvements. One well-known example is the Pilbara Iron Ore Mine in Western Australia, where staff members remotely control vehicles and machinery from a central hub close to Perth [67]. Applications of autonomous haulage systems (AHS) and automated drilling systems (ADS) have significantly improved efficiency and safety in the mining industry [68]. Ellem [69] and Tinto [70] have demonstrated that these systems maximize mining operations by minimizing human intervention. At Jimblebar mine, using more than 50 AHS units reduced drilling costs by 40%, increased production by 25% and reduced accidents by 80% [71]. Fortescue Metals Group (FMG) installed 183 AHS units at its Solomon and Chichester mines, thus reducing production costs by 50% [72], and Rio Tinto installed 130 AHS units at Pilbara Iron Ore Mine located in Western Australia, improving operational performance by 11% [73]. Boliden uses 11 Komatsu FrontRunner AHS units at Aitik copper mine in Sweden [74]. Since these systems require substantial initial investment and robust infrastructure, smaller mining companies face challenges with accessing them [75].

Drone-based swarm technologies also enhance mining exploration. Emesent drones, for example, support real-time 3D mapping at locations such as Olympic Dam (Australia) [76], Kiruna (Sweden) [77], and Las Cuevas Caves (Belize) [78] as part of the DARPA Subterranean Challenge [79]. They do face limitations like short battery life, signal interference, and decreased accuracy in muddy conditions [80,81,82], and environmental factors often exacerbate these constraints in subterranean settings [83,84]. However, drones still offer important advantages, for instance, increased accessibility and improved safety when operated in small groups [80,81,82]. Similarly, Ascot Resources (Canada) [85] and Chelopech Mine (Bulgaria) [86] employ Exyns A3R drones that use LiDAR and SLAM for real-time mapping, providing greater operational flexibility in difficult circumstances. Even so, dust and moisture, coupled with short battery life, can impede their effectiveness in harsh environments [87,88]. By automating mineral extraction at mines like Williams (Canada), Pyhasalmi (Finland), and El Teniente (Chile), Sandvik’s AutoMine technology has improved productivity, safety, and economic outcomes [89,90,91]. Its use at the Syama Mali Solutrecan gold mine has lowered labor costs and enhanced efficiency [90]. Nonetheless, reliance on centralized control infrastructure and steep deployment costs have limited their broader adoption [92].

As for research and development, the Horizon 2020-funded UNEXMIN project uses UX-1 robots to automatically map and scan flooded mines to reopen 30,000 closed sites in Europe for sustainable exploration [93,94,95,96]. Even in complex underwater environments, these robots can map a large area. Notwithstanding their success, the UX-1 robots have faced challenges such as signal attenuation in turbid water and energy limitations during extended operations [97,98]. Similarly, Epiroc’s NEXGEN SIMS project uses swarms of self-governing robots to drill, inspect and move around in mines, thus lowering the risks to humans [99]. But dependability of these swarms of robots is impacted by rockfall, dust and thermal interference [100]. NASA’s Swarmies and RASSOR robots have extended the use of swarm robotics technology to mining on other planets. On the Moon and Mars, RASSOR robots were used to harvest regolith [101,102,103], and Swarmies were developed for in situ resource utilization on Mars [104,105,106]. Despite their lightweight and modular design, these robots’ scalability and mechanical deterioration under abrasive conditions continue to be issues for their reliability in terrestrial mining operations [102]. Table 2 summarizes the applications of swarm robotics in the mining sector, its advantages and mining-specific limitations.

Swarm robotics in mining is currently at a developing phase, and detailed discussions on case studies from both surface and underground operations with their applications are presented in the paragraph that follows. Even though swarm robots can assist with safety and logistical issues in mining, significant mechanical advancements are required for the actual excavation process, which includes environmental control, tunnelling and drilling, to achieve higher efficiency of mining. Under such circumstances, biomimetic designs can significantly reduce the wear and damage to instrumentation and save energy. The next section will look at how biomimicry-based engineering techniques could increase the sustainability and productivity of mining.

### 3.3. Critical Limitations and Failure Modes of Swarm Robotics

Despite its potential to automate mining and boost operational efficiency, swarm robotics has encountered many difficulties. Communication reliability is a crucial issue, because decentralized swarm coordination in underground conditions is frequently hampered by dust, humidity and tunnel obstructions [87,100]. Environmental hazards like rockfalls, mechanical vibrations and extreme temperatures can impact sensor performance and mechanical reliability of robots [87,98,100]. Energy-related problems, particularly the short battery life of drones and autonomous vehicles, limit their operating time in remote or underground locations due to limited charging stations [80,97]. Additionally, the inability to standardize the systems makes their integration more challenging, and scalability and interoperability pose challenges since large swarms need a lot of processing power to coordinate in real time. Finally, high capital expenditures [75] that involve significant investments in hardware infrastructure are challenging, especially for small mining companies.

Above-mentioned communication failures, energy shortages and environmental hazards are some of the challenges that could reduce swarm robot’s productivity in challenging and dynamic mining environments. Furthermore, as mining operations become more complex, interoperability and scalability remain major obstacles. Section 5 further discusses potential applications of these integrated solutions in the mining industry to make it safer, more sustainable and more efficient.

## 4. Biomimicry-Based Technologies

### 4.1. Overview of Biomimicry Principles

**Figure 3:**
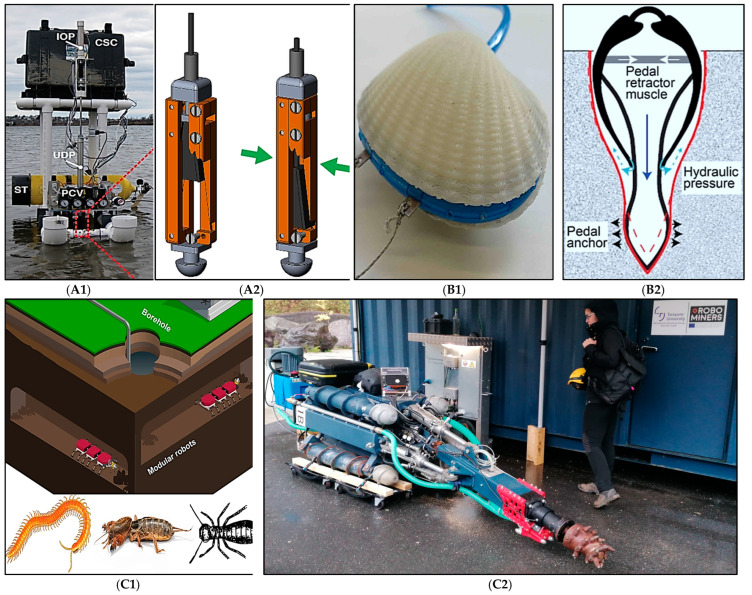

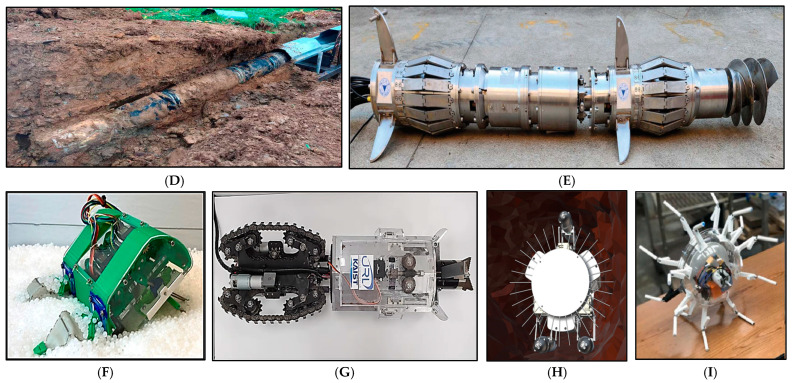
Examples of biomimetic designs and technologies in mining. (**A1**) RoboClam full prototype [110] (Image courtesy of IOP publisher, CC0) and (**A2**) its CAD design [111] showing an internal cutaway of the sliding wedge linkage. The green arrows in CAD highlight the contraction of the end effector, which fluidizes the surrounding sediment by mimicking the Atlantic razor clam’s rapid valve movements. Key components include in–out piston (IOP), control system case (CSC), up–down piston (UDP), scuba tank (ST), and pressure control valves (PCV). (**B1**) Actuated Bivalve Robot full prototype with 3D printed shell [112] (Image courtesy of Elsevier publisher) and (**B2**) its schematic diagram [21] showing bivalve’s pedal retractor muscle, pedal anchor, and hydraulic pressure mechanism that collectively fluidize the sediment for effective burrowing. The large downward arrow indicates the force generated by the pedal retractor muscle pulling bivalve deeper into the sediment, while the small outward arrows represent the hydraulic pressure expanding the foot to create an anchor within the substrate. (**C1**) ROBOMINERS schematic diagram using centipede, mole cricket, and termite-inspired modular robots for underground mining operations [113] and (**C2**) ROBOMINER robot at Mezica mine, Slovenia (Image courtesy of the UNEXUP project). (**D**) BADGER design is inspired by inchworm using anchor-pull [114] and anchor-push mechanisms to traverse underground environments [115] (Image courtesy of John Wiley and Sons publisher). (**E**) Stratloong inspired by earthworm’s peristaltic motion for underwater exploration, with retrograde wave and segment elongation for soil penetration [116] (Image courtesy of IEEE publisher). (**F**) EMBUR pacific mole-crab prototype, increasing burrowing capacity through power strokes [117]. (**G**) Mole-inspired Robot African naked mole rat prototype, featuring a drill bit and forelimb structure to enhance excavation and soil removal [118] (Image courtesy of the KAIST and Prof. Hyun Mung). (**H**) Whisker-inspired navigation system modelled after the sensory abilities of rodents and naked mole rats, utilizing hall effect sensors and whisker arrays to map underground terrain [119] (Image courtesy of MDPI publisher, CC0). (**I**) Golden Wheel Spider-inspired rolling robots, employing wind-assisted and pendulum-driven locomotion for energy-efficient movement across desert and planetary terrain [120] (Image courtesy of Elsevier publisher).

Biomimicry provides creative and sustainable solutions to engineering problems by leveraging natural patterns and processes [107]. Applying biomimicry techniques in tasks ranging from mapping to exploration and excavation has transformed the mining sector. Designed to reduce mining’s environmental footprint, raise operational efficiency, and adapt to shifting conditions [108,109], these technologies are examined here with an emphasis on practical applications, major advantages, and inherent drawbacks. As is illustrated in Figure 3, diverse biomimetic innovations and designs are making significant impact within the mining sector.

Mining environments pose specific mechanical, chemical, and thermal challenges. Even though biomimetics has influenced many fields, including materials science and soft robotics, its application in mining is still limited. For instance, simulating biological adaptations for burrowing can lower energy consumption and friction in hard rock drilling, and rodent-like whisker systems can improve threat detection in underground passageways that are dusty or have poor visibility. Section 4.2 examines biomimetic designs that particularly deal with resource extraction, mining excavation, and environmental recovery.

### 4.2. Core Biomimicry-Based Technologies in Mining Contexts

Biomimicry has influenced several robotic mining excavation and exploration systems based on natural models such as termites, clams, crabs, spiders, earthworms and moles [21]. Biomimetic designs offering creative drilling and excavation solutions include RoboClam and bivalve robots. RoboClam uses 90% less energy when excavating in damp soils compared to traditional methods, because it mobilizes material by contracting its valves, which is a technique inspired by the Atlantic razor clam [110,111,121,122,123,124]. Likewise, by mimicking the digging behavior of bivalves through rocking motion and water displacement, bivalve robots reduce digging forces by seven times [112,125]. Although these designs have many advantages, they are limited to specific soil conditions. For example, bivalve and RoboClam robots are best suited for soft moist soils and have limited scalability for hard rock mining [110,112]. The ROBOMINERS project was inspired by centipedes, termites and mole crickets. It can dig underground without a GPS and is suitable for mining deep mineral deposits, rehabilitating mines and reopening abandoned sites [113,126,127]. However, due to their highly specialized and complex nature, integrating these robots with existing mining systems is challenging [126]. Similarly, CRABOT, a mole crab-based platform, increases digging efficiency by 50%, by utilizing its burrowing appendages [117,128,129]. However, lack of investigations on its performance in hard rock mining environments limits its applicability [129].

Biomimetic robots have revolutionized mapping and mining exploration operations. Mole-Bot and Rescue Mole are two examples of robots that successfully dig and move underground by imitating their natural prototype’s burrowing mechanisms [130,131,132,133,134,135,136,137]. However, these designs are not suitable for high-pressure or abrasive environments, where their mechanisms may become inefficient and wear down quickly [138,139]. Based on the peristaltic motion of earthworms, Stratloong effectively penetrates soil with a motion efficiency of over 90% [116]. Despite its success in seafloor exploration, its effectiveness decreases in rocky or non-cohesive soils. The BADGER robot and NASA’s inchworm-inspired designs allow for precise tunnelling through successive anchoring and extension [115,140,141,142]. Thus, these systems have speed limitations and are prone to mechanical wear in abrasive mining environments [143]. The RM3 robot and biomimetic whisker sensors (BMWS) are examples of whisker-inspired technologies that enhance tactile perception and navigation in difficult terrain by mimicking the senses of rodents [119,144,145]. Despite their effectiveness in mapping and object detection, these robots and sensors perform poorly in extremely dusty or humid conditions that are common in mining operations [144]. Mobility systems inspired by the golden wheel spider provide energy-efficient exploration capabilities in difficult terrain. Using wind-assisted locomotion and pendulum-driven motion, rolling robots can navigate efficiently [120]. However, they cannot work well on steep and uneven terrain; therefore, they are less useful for most mining scenarios [146]. Table 3 summarizes the key biomimetic mining-applicable innovations along with their applications and mining-specific limitations.

Mining productivity, safety, and sustainability stand to benefit from biomimetic drilling or tunnelling technologies, NIAs, and swarm robots, particularly when these three strategies are combined to manage energy usage, ensure robust logistics, and maintain dynamic adaptability. In the following section, the integrated approach that connects them all is examined.

### 4.3. Critical Limitations and Failure Modes of Biomimicry-Based Technologies

Despite their potential to offer solutions, significant energy efficiency or decreased excavation forces, many biomimetic technologies are still limited to soft soils or laboratory-scale settings, which prevents their widespread adoption. Mechanical robustness of these technologies is of paramount importance, as many bio-inspired robots are not designed to survive high pressures, abrasive rocks or other mechanical shocks characteristic of deep mines [110,129,138,139,143]. Besides these, other aspects as well specific to a certain biomimetic technology can serve as hurdles for its wider application. For example, the speed of peristaltic or rocking motions of RoboClam usually lags that of traditional drilling techniques [110,112,116], and its integration with widely used mining equipment can be costly, requiring extensive retrofits or specialized attachments. Unless performance gaps are addressed by applying advanced materials or hybrid drilling techniques, this disparity might prevent widespread adoption of the technology. Furthermore, achieving dependable extended operations is further hampered by sensor degradation, particularly for whisker-based detection in highly dusty or humid environments [144,145]. These examples demonstrate that although biomimicry-based technologies might be highly promising for achieving more energy-efficient and environmentally friendlier mining, their application to challenging underground environments requires further mechanical and material advancements. Therefore, although biomimicry has a huge potential to improve the mining sector, it is not yet sufficiently robust to handle the challenges that modern mining operations involve. Because it relies on translating biological principles into functional engineering solutions, it might face challenges with scalability, accuracy and integration when applied in complex mining environments. Section 5 presents comprehensive discussions on how these integrated approaches could help create mining environments that are more robust, effective and sustainable.

## 5. Systematic Integration and Applications in Mining

Leveraging the resource efficiency of biomimicry-based solutions, the decentralized robustness of swarm robots and the optimization capabilities of NIAs, we present a unified framework that builds on Section 2, Section 3 and Section 4. From long-term sustainability through biomimetic reclamation techniques to real-time routing in the face of geological uncertainty, this combined performance may be able to address fundamental mining issues. Section 5 builds on the discussions from previous sections to propose integrated solutions for further improving various aspects of mining operations by incorporating biomimicry-based models and technologies with a focus on better sustainability, scalability and adaptability.

### 5.1. Key Challenges in Modern Mining and Integration Potential

The mining industry faces several interrelated challenges in addition to the limitations discussed under Section 2, Section 3 and Section 4. These include energy inefficiencies, drilling difficulties, navigating in hazardous environments, logistical inefficiencies, safety risks, environmental impacts, issues with adaptability in changing conditions and scaling limitations. Table 4 summarizes these challenges and relates them to specific contributions that swarm robotics, NIAs and other biomimicry-based technologies could bring.

### 5.2. Integrated Solutions to Mining Challenges

Combined applications of NIAs, swarm robotics and other biomimicry-based technologies could offer efficient solutions to complex problems facing the mining sector, as presented in Table 4. These solutions could improve mining’s efficiency, safety and sustainability by combining the best features of each method/technology. This section presents comprehensive discussions on such integrated solutions with an emphasis on how these technologies would function in actual mining applications.

#### 5.2.1. Energy Consumption

NIAs + Biomimicry-Based Technologies: Drilling operations in hard rock mining often struggle with excessive wear, overheating, and high energy consumption of equipment [147]. Drawing on the burrowing mechanism of Atlantic razor clams, biomimetic designs such as RoboClam reduce drag and thereby lower energy usage during penetration [22]. While initially developed for soft sediments, the fluidization approach of RoboClam can be adapted to stabilize pre-drilling tasks in hard rock environments like granite or basalt, reducing friction and tool wear. Additionally, real-time geological feedback can guide PSO in fine-tuning parameters such as torque, speed, and pressure, thus preventing overheating and maintaining steady penetration rates [28]. By accounting for transitions across different rock types—for instance, moving from quartzite to shale—this method fosters more efficient energy utilization, less drill bit wear, and extended tool life [148].

Rio Tinto’s Diavik Diamond Mine in Canada is known to face significant challenges relating to energy efficiency and tool durability, especially when drilling through complex geological formations [149,150]. Conventional drill bits often overheat, necessitating frequent replacements and extending downtime. Utilizing technology inspired by RoboClam could help fluidize surrounding materials and reduce drag during drilling, potentially resulting in energy saving and decreased mechanical wear. Moreover, the application of PSO allows for dynamic modification of drilling parameters, such as penetration, torque, and speed, to adapt to changing geological conditions. This integration would enable the mine to reduce energy consumption, extend tool life, and lower overall operating costs. The combined use of biomimicry-based technologies and NIAs would provide innovative solutions to address energy-intensive mining challenges, particularly during exploration, core logging and production drilling stages.

#### 5.2.2. Navigation and Mapping

NIA + Swarm Robotics: Accurate navigation and mapping are essential for resource assessment, hazard detection and infrastructure planning in complex and hazardous sites such as underground mines, flood zones and toxic areas. Using Simultaneous Localization and Mapping (SLAM) technology, swarm robotic systems such as Emesent drones [76,77,78,79,85,86] and UX-1 underwater robots [93,94,95,96] can navigate these challenging environments on their own and create complex 3D maps. ACO enhances this capability by merging and refining data from several robots into a single high-precision global map, drawing inspiration from the pheromone-tracking behavior of ants [31].

At Australia’s Olympic Dam, one of the largest underground mines in the world, the management of an extensive network of tunnels and the identification of mineral reserves heavily depend on accurate mapping and navigation. Traditional mapping methods are time-consuming and pose safety risks, especially in the presence of complex geological features [151]. Autonomous mapping and navigation by drones equipped with SLAM technology are being explored for mapping large underground areas [152,153]. UX-1 robots could also be applied to assess the structural stability of flooded areas and locate undiscovered mineral resources. Integrating data from multiple drones and robots into a single comprehensive 3D map using ACO will help eliminate data redundancy and enhance accuracy. This approach would improve situational awareness and resource identification while significantly reducing the time and risks associated with manual mapping. By improving planning and operational efficiency, such techniques could contribute to safer and more efficient mining operations.

#### 5.2.3. Transportation and Logistics

NIA + Swarm Robotics + Biomimicry-Based Technologies: Mining productivity depends heavily on efficient logistics and transportation, particularly in large open-pit and underground operations. AHS [67,68,69,70,71,72,73,74] and Sandvik AutoMine [89,90,91] are two examples of cutting-edge technology applications, were fleets of self-sufficient loaders and haul trucks function with little help from humans. Modular vehicle concepts based on moles improve maneuverability in steep and rugged terrain [130,131,132,133,134,135,136,137]. The vehicle can freely change its body configuration while remaining stable by integrating these designs into segmented chassis. Even on slippery or uneven surfaces, modern traction technology with a variable tread pattern ensures steady grip. These characteristics are comparable to the mole’s innate capacity to move successfully under harsh conditions. By examining real-time data on traffic patterns, topography and equipment availability, PSO [154,155] further optimizes haul routes to provide effective and dynamic route management.

Open-pit coal mining operations in Australia face substantial logistical challenges in managing the daily transportation of millions of tons of ore, especially during rainy season when loose rocks and mud increase the risks of equipment slippage and damage [156,157]. Underground mining operations also have wet road conditions, which increase hazards such as wheel slippage for transport trucks [158,159]. A potential solution could be provided by biomimetic modular vehicle designs inspired by mole movement, which would improve adaptability to challenging terrains. Vehicles like AHS and Sandvik AutoMine, with articulated parts and adjustable traction mechanisms, can distribute weight evenly and maintain an optimal grip on wet surfaces or steep slopes. Real-time haul route optimization, supported by a PSO algorithm, can further adjust routes dynamically based on weather, traffic patterns, and equipment conditions. For instance, during periods of heavy rainfall, the algorithm could prioritize routes with better drainage to maintain operational continuity and safety. Integrating NIAs, swarm robotics, and biomimetic modular designs would significantly enhance the resilience and reliability of the transportation system at mine sites while streamlining logistics. These advancements would enable maintaining high production levels while reducing operational and environmental risks in large-scale mining operations.

#### 5.2.4. Safety and Hazard Management

Swarm Robotics + Biomimicry-Based Technologies: Safety remains of paramount importance in the mining industry, especially in regions susceptible to earthquakes, rockfalls and gas leaks. The whisker sensors on the RM3 robots and BMWS are an example of a biomimicry design that replicates a rodent’s tactile sensitivity to identify stress variations and minute movements in the rock mass [119,144,145]. These sensors allow operators to stay ahead of potential threats by generating early warning signals. In seismic zones or during blasting operations, these sensors detect rock collapse or gas leakage precursors and monitor the redistribution of stress. Swarm robotics allows for distributed risk monitoring, which improves hazard management. A network of robots with whisker sensors could cooperate to monitor strategic locations, to guarantee comprehensive coverage and real-time data collection. For example, a swarm of robots could detect micro-seismic changes during staged caving operation and analyze stress variations in roof supports and pillars for underground stability and safety monitoring. Through improved predictive maintenance and decreased worker exposure to hazardous areas, this integration could substantially improve the overall safety at the mine.

At Chile’s Chuquicamata mine, the shift from open pit to underground mining has introduced challenges like seismic activity and stress redistribution [160]. Micro-seismic monitoring in Shirengou Iron Mine has revealed issues, such as rock damage during transitions [161]. Continuous geostress monitoring in rock pillars, underground tunnels, fracture zones, and transition areas could be achieved using RM3 robots or BMWSs equipped with whisker-style sensors. Distributing these tasks among multiple robots through swarm robotics creates a dependable safety system that can rapidly detect and address hazards. Such integration dramatically lowers collapse risks and accidents, thereby enhancing workplace safety.

#### 5.2.5. Environmental Impact

Swarm Robotics + Biomimicry-Based Technologies: Minimizing the environmental impact of mining operations requires creative solutions that minimize geological disturbance while maintaining mining efficiency. Inspired by the peristaltic motion of earthworms, the biomimetic tunneling systems’ circulators, such as Stratloong [116], BADGER and NASA Inchworm robot [115,140,141,142], offer a new approach to subsurface excavation. These methods use successive anchoring and extension operations to create high-precision tunnels that reduce vibrations and stresses on the surrounding rock mass. This method ensures that the integrity of the formation is maintained by avoiding extensive fracturing and subsidence that often accompany traditional drilling and blasting procedures. Earthworm-like and inchworm-like designs make these technologies particularly appropriate for environmentally sensitive areas, such as those near water-bearing underground layers or sites that require ecological protection. NEXGEN SIMS robots use swarm robotics principles, in addition to this tunneling technique, to operate effectively within carefully constructed tunnels, performing recovery and surveillance tasks [99]. Although NEXGEN SIMS is not using peristaltic motion, its modular and autonomous capabilities allow it to move efficiently, assess its environment and perform coordinated tasks.

In Sweden, Boliden mine transitioned its operations underground to minimize environmental impacts at the surface [162]. In this context, the Stratloong robot, inspired by the peristaltic motion of earthworm, could be employed for precise excavation of tunnels with minimal disturbance to the surrounding rock mass. This biomimetic method would help maintain the integrity of the underlying geology by reducing stress and vibrations typically associated with traditional tunnel excavation methods. Following tunnel construction, deploying NEXGEN SIMS swarm robots equipped with advanced environmental sensors could facilitate tasks such as targeted revegetation, soil stabilization, and tunnel stability monitoring. These robots could collaborate to address potential ecological issues linked to mining operations while minimizing environmental impacts. By combining the precision of the Stratloong robot with the adaptability and efficiency of NEXGEN SIMS robots, Boliden mine could set a benchmark for environmentally friendly mining practices while maintaining operational efficiency and compliance with stringent environmental regulations.

#### 5.2.6. Adaptability in Dynamic Environments

NIA + Swarm Robotics + Biomimicry-Based Technologies: In dynamic mining environments, especially in flooded or unstable mines, unexpected issues like water inflow, equipment failure or geological changes require adaptable and efficient solutions. By employing biomimetic technology, the UX-1 robots can maneuver through challenging environments, because they move like fish, which makes them stable and nimble underwater [93,94,95,96]. The robots integrate global data and optimize their routes using the ACO technique. ACO ensures efficient pathfinding, by constructing a virtual map with digital pheromones that show each path’s quality based on real-time feedback such as water flow, structural stability and mineral density [45,46,47]. High-value regions are being prioritized, and unnecessary exploration is prevented by integrating all data into a single representation. The GWO algorithm, which dynamically allocates tasks like hazard identification, resource mapping and structural analysis, is used to improve the ant colony’s work allocation in the interim. To facilitate real-time coordination and ensure seamless operation even in the face of unforeseen circumstances, GWO ranks targets based on the mine’s changing conditions, drawing inspiration from the grey wolf’s hierarchical hunting style [32]. By combining ACO and GWO, UX-1 robots can dynamically locate, map, and assess resources while preserving operational safety and precision.

Flooding and dynamic mining conditions, such as those encountered at Idrija Mine in Slovenia and Kiruna Mine in Sweden, are known to present significant challenges [163,164]. In underwater tunnels, the UX-1 robot, inspired by fish movement, could provide stability, while ACO would optimize exploration paths to avoid hazards like unstable sections and prioritize mapping of high-value locations. The integration of these technologies could generate precise 3D maps that highlight resource locations and structural flaws, facilitating strategic reopening of critical sections. Similarly, Kiruna Mine regularly encounters flooding and landslides that disrupt operations and compromise workers’ safety [165]. The UX-1 robot, guided by ACO, can dynamically adjust its course in real time to ensure thorough investigations while avoiding hazards. GWO can further enhance collaboration among robots by assigning tasks such as avoiding collapsed areas or focusing on mineral-rich zones. This integrated approach would allow the mines to maximize resource recovery, enhance safety, boost production, ensure operational continuity under challenging situations and manage unpredictable conditions.

#### 5.2.7. Scalability and Cost-Effectiveness

NIA + Biomimicry-Based Technologies: Implementing state-of-the-art mining technology can be challenging due to associated costs and scalability issues, especially in pilot projects or businesses with limited funding. Inspired by the efficient burrowing system of mole crabs, CRABOT is meant to be implemented gradually in compliance with project specifications. Each CRABOT unit is lightweight, small, and allows for exceptional maneuverability in a variety of mining environments, such as loose gravel or compacted soils [117,128,129]. This modularity allows operators to scale their operations efficiently, adding units as demand rises without the need to make large upfront investments. By reducing component wear and energy consumption, the robot’s design also reduces operating and maintenance costs. PSO, which optimizes task scheduling and dynamically distributes resources [37,38,39], can improve CRABOT’s capabilities. To boost productivity while decreasing waste and downtime, PSO looks at operational and geological data such as excavation priority, equipment availability and ore grade. This integration would help to create a mining system that is both scalable and reasonably priced by ensuring effective operation of each CRABOT unit.

At Ecton Mine in the UK, a small-scale pilot project faces challenges stemming from limited funding and the need for cost-effective solutions [166]. The CRABOT system, with its lightweight and modular design, could allow for efficient excavation in confined spaces. PSO can help identify high-priority excavation sites and allocate tasks based on real-time performance data. For instance, PSO can recommend deploying additional CRABOT units in areas with higher ore concentrations, potentially maximizing recovery and redirecting resources from less lucrative zones. As operations expand and new reserves are discovered, the scalability of CRABOT systems would allow for a seamless transition to full-scale mining. This integration would significantly reduce operating costs and investment risks and facilitate economically sustainable mining practices.

### 5.3. Summary of Integrated Solutions

The problems and solutions discussed under Section 5.2 provide a comprehensive review of how NIA, swarm robotics, and biomimetic technologies can be integrated and adopted, combining their attributes to enhance mining activities and address mining related problems. The comprehensive analysis has been summarized in Table 5.

Table 5 illustrates in summary how the integrated solutions combine the capabilities of NIAs, swarm robotics, and other biomimicry-based technologies to solve a range of mining challenges individually. In this table, the rows pertain to a specific mining challenge and the columns denote how NIAs, swarm robotics, and other biomimicry techniques can help to address it. A tick in a column implies that the technique is used in overcoming that challenge. For instance, the Energy Consumption row indicates the impact of combining both NIAs and biomimicry designs, with clam-inspired mechanisms for mitigating energy consumptions for excavation, while combining PSO-with drilling processes brings further improvement allowing the system to become more effective. In the Navigation and Mapping row, the two NIAs approaches are combined with swarm robotics, where drones and UX-1 robots perform the mapping and Ant Colony Optimization (ACO) converts the captured data into accurate real-time maps. Also, in Transport and Logistics, the table shows the supporting roles of NIA, swarm robotics, and biomimicry, through mole inspired designs which greatly improve the system’s ability to control mining fleets. The Safety and Hazard Management, Environmental Impact, Adaptability in Dynamic Environments, and Scalability and Cost-Effectiveness rows follow the same structure, with their integration column describing how each technology interfaces with the others to offer an integrated solution. This illustrative summary reinforces the claim that the table is not a mere catalogue of technologies, but instead portrays how these technologies can be harnessed together to achieve enhanced efficiency, safety, and sustainability in mining operations.

## 6. Conclusions and Recommendations

This study presented a comprehensive analysis on the revolutionary potential of swarm robotics, biomimetic technologies and NIAs in addressing pressing issues facing contemporary mining operations. When combined, these methods could provide highly effective ways to improve the mining sector in terms of safety, environmental impact, cost-effectiveness, energy consumption and real-time adaptability. Better resource allocation, scheduling and pathfinding, as well as proficiency in multi-objective optimization, could be made possible by NIAs, while biomimetic technologies, using biological systems as inspiration to develop highly effective, lightweight and energy-efficient designs, could improve drilling, tunneling and hazard detection. For mapping, exploration and logistics, swarm robotics could offer scalable decentralized coordination, especially in remote or dangerous settings. When combined or integrated, these technologies could achieve substantial advances in mining, resulting in more flexible, sustainable and profitable mining operations. However, there are hurdles that still must be overcome before these ideas can be applied in the mining industry. Real-time decision-making in dynamic environments, communication dependability and scalability are important aspects that must be addressed. Engineering designs of biomimetic technologies must be further developed into reliable mechanical systems for industrial-scale field applications in actual mining operations. Interdisciplinary research and development are key in achieving the full potential of these technologies and their successful field applications. It is suggested that the development of scalable and modular biomimetic systems that could operate reliably in a variety of geological settings and on an industrial scale be the first major milestone in achieving this goal. Enhancing real-time intelligence via artificial intelligence (AI)-powered swarm robotics and enhanced NIAs could facilitate dynamic decision-making and increased environmental adaptability. Mining systems’ sustainability and operational efficiency could be improved by tackling energy challenges with sustainable solutions such as solar technologies. Cost-cutting measures such as creating reliable and affordable prototypes would be essential for promoting wider adoption, especially by small and medium-sized mining companies or operations.

It is also suggested that establishing ethical and regulatory frameworks is essential to ensure that the use of autonomous systems in the mining industry complies with safety, social and environmental requirements. Achieving the future goal of more intelligent, flexible, sustainable, safer, socially conscious and environmentally friendlier mining operations that meet the changing needs of the sector could be made possible by the combined applications of NIAs, biomimicry-based technologies and swarm robotics. Furthermore, investigating applications and the associated resilience and scalability needs of these methods and technologies for deep-sea mining, extraterrestrial mining and mining in other harsh environments could open a new era for the mining sector.

## Figures and Tables

**Table 1:** Core NIAs in Mining: Key Characteristics, Advantages and Limitations.

NIAs (Bio-Inspired)	Mining Applications	Advantage	Mining-Specific Limitations
PSO [Bird/fish flocking]	Exploration (stope identification) [37]; Mine Planning (block scheduling) [38]; Logistics (truck dispatch) [39]; Safety/Env. Monitoring (hazard detection, vegetation indices) [40].	Relatively few parameters, quick convergence, suitable for both discrete and continuous data.	Tends to converge prematurely if swarm diversity is lost; large block models can stress computational resources; real-time updates under changing geology can be challenging without local autonomy [37,38,39,40].
ABC [Honeybee foraging]	Exploration (kriging variance reduction), Resource Management [29,41].	Balanced exploration–exploitation, good multi-objective performance.	Less tested under large-scale, high-dimensional mining models; hive-size/colony parameter tuning can be tedious; lacks robust adaptation to real-time production changes [41].
GOA [Grasshopper swarming]	Exploration (mineral zone delineation), Resource Management [42].	Works well in continuous search, fewer hyperparameters than some other swarm methods.	Limited industrial validation in complex mine settings; may stall without hybridization or local heuristics; sensitivity to abrupt geological or operational shifts [42].
BA [Echolocation of microbats]	Exploration (mineral mapping) [43], Safety/Risk (fault detection) [44], Environmental [44].	Effective balance of local/global search; handles dynamic data well.	Pulse rate, loudness parameter tuning is non-trivial; rarely tested in full-scale mines; real-time usage may require synergy with decentralized control to handle rapid condition changes [43,44].
ACO [Ant pheromone-based foraging]	Mine Planning (route optimization, production scheduling) [45,46,47], Safety/Risk (escape routes) [48].	Excellent for discrete/graph-based tasks, robust adaptation to environmental changes.	Slow convergence on high-dimensional or multi-level block models; pheromone settings can be tricky; decoupled approaches often needed for large mines [45,46,47,48].
GWO [Grey wolf pack hierarchy]	Long-Term Scheduling (NPV maximization) [18,49], Safety (fault detection) [50].	Fewer parameters than some swarm algorithms, good exploration–exploitation balance.	Limited real-world case studies at industrial scale; local minimal risk in extremely complex geological or operational spaces; demands distributed data handling for real-time updates [18,49,50].
FA [Light intensity-based firefly attraction]	Logistics Optimization (equipment dispatch), Scheduling [51].	Handles multi-modal functions; relatively straightforward to parallelize.	Prone to local minimum if brightness/absorption parameters are off; large-scale scheduling may outstrip typical runtime constraints; lacks established real-time adaptation protocols [51].
SSA [Salp swarm chain formation]	Safety/Risk (blasting vibration prediction, hazard mapping) [52].	Simple leader–follower mechanics, can achieve solid global exploration.	Not broadly tested across all mining tasks; boundary conditions and chain-model parameters can hamper performance under dynamic mine layouts; heavily reliant on local sensing/communication [52].

**Table 2:** Core Swarm Robotics in Mining: Key Characteristics, Advantages and Drawbacks.

Robotics Approach [Project]	Mining Applications	Advantage	Mining-Specific Limitations
AHS [Rio Tinto, FMG, BHP, Sandvik]	Mine transportation (Autonomous haul trucks for ore transport) [67,68,69,70,71,72,73,74].	Increase production, reduce costs and accidents.	High initial investment requires robust infrastructure, less flexibility [75].
Hovermaps [Emesent]	Mine exploration (3D mapping drones for underground mines) [76,77,78,79,83,84].	3D autonomous scanning reduces human exposures to dangerous areas.	Battery and signal constraints in deep flood tunnels [80], turbid water degrades sensor accuracy [81], complex multi-robot data coordination [82].
Exyns A3R [Exyns Technology]	Mine exploration (3D mapping in underground mines) [85,86].	Uses LiDAR and SLAM for real-time data collection.	Short battery life [80], dust and humidity degrade sensors [87], no integrated cargo capacity for excavation tasks [88].
AutoMine system [Sandvik]	Surface and underground mining (Tele-remote control of loaders and haul trucks) [89,90,91].	Automates extraction, improves productivity and safety.	High deployment costs [75], requires centralized control infrastructure [92].
UX-1 [UNEXMIN project]	Mine exploration (Mapping and exploring flooded mines) [93,94,95,96].	Autonomous mapping, environmental monitoring.	Battery life limitations during extended missions [97], signal degradation in turbid water affects data accuracy, coordination complexity with large swarms [98].
NEXGEN SIMS Autonomous Robots [Epiroc Project]	Surface and underground mining (Autonomous transportation, drilling, and inspection) [99].	Reduces human intervention, enhances safety.	Rockfalls, dust, and heat can disrupt sensors and communication links, and lack of standardized systems across mining layouts [100].
RASSOR * (* updated name: IPEx) [NASA]	Lunar and Martian mining (Regolith excavation and processing on the Moon and Mars) [101,102,103].	Lightweight and modular designs, distributed excavation tasks.	Limited Earth-based field demonstrations, dust infiltration and mechanical wear in typical mines untested, no real-time data on scaling to multiple diggers in large open-pit or underground scenarios [102].
Swarmies [NASA’s Swarmathon Competition]	Space exploration (Resource collection for ISRU tasks in Mars missions) [104,105,106].	Adaptive route updates, local decision-making reduces global overhead.	Primarily tested in controlled NASA contexts, unproven reliability in dusty, narrow mine passages, hardware durability unknown for abrasive underground conditions [104,105,106].

* Previously named RASSOR (Regolith Advanced Surface Systems Operations Robot), IPEx (In-Situ Resource Utilization Pilot Excavator) is NASA’s robotic system developed for automated excavation and reutilization of lunar surface material [101,102].

**Table 3:** Core Biomimetics in Mining: Key Characteristics, Advantages and Drawbacks.

Robots (Bio-Inspired)	Mining Applications	Advantage	Mining-Specific Limitations
RoboClam (Atlantic Razor Clam)	Surface mining, drilling (Contracts valves to fluidize soil, reducing drag) [110,111,121,122,123,124]	Significant energy savings (up to 90%) in soft soil environments, reduces drag during drilling	Limited to soft soil; untested scalability for hard rock conditions [110].
Actuated Bivalve Robot (Bivalve)	Surface mining, drilling (Rocking motion and water expulsion to fluidize sediment) [112,125]	Reduces required force for sediment penetration, efficient in soft sediment environments	Reduced efficiency in compacted or dry soils, limiting its broader applicability [112].
ROBOMINERS (Mole Crickets, Termites, Centipedes)	Underground mining, mine rehabilitation (Autonomous navigation and excavation without GPS) [113,126,127]	Environmentally friendly and precise excavation, ideal for deep mining and abandoned sites	Integration into traditional mining workflows is challenging, operational stability in complex environments is untested [126].
CRABOT, EMBUR (Mole Crab)	Underwater mining, excavation (Power strokes with appendages to improve excavation) [117,128,129]	Enhances excavation efficiency by 50%, lightweight and adaptable for narrow spaces	Effectiveness in hard rock or high-pressure environments remains unproven [129].
Mole-Bot (Mole)	Mine excavation for shallow deposits (Mimics forelimb and incisors for digging) [130,131,132,133,134,135,136,137]	High precision in shallow digging tasks, effective debris removal	Struggles in abrasive or high-pressure environments, limited performance in deep or compact deposits [138,139].
Stratloong (Earthworm)	Seabed exploration, underwater mining (Peristaltic motion to penetrate strata) [116]	High motion efficiency (over 90% in tests), minimizes environmental disturbance	Limited effectiveness in unstable or non-cohesive soils; sensitive to mechanical wear during extended operations [116].
BADGER, NASA Inchworm (Inchworm)	Underground tunneling, excavation (Sequential anchoring and extension for tunneling) [115,140,141,142]	Capable of precise tunneling and curved path navigation, minimizing excavation disruption	Limited speed and durability under abrasive conditions; constrained adaptability for irregular or unpredictable paths [143].
RM3 Robot, BMWS (Rodents)	Underground mining, hazard detection (Mimics whisker sensing for navigation) [119,144,145]	Highly accurate object detection and mapping in low-visibility environments	Sensor accuracy degrades under extreme humidity or high dust concentrations [144].
Spider Rolling Robot (Golden Wheel Spider)	Surface mining, exploration (Wind-assisted rolling for efficient movement) [120]	Energy-efficient movement on flat or desert-like terrain, lightweight design	Ineffective on steep or highly irregular terrains; limited operational range in adverse weather conditions [146].

**Table 4:** Overview of Mining Challenges and Relevant Technology.

Mining Challenges	Mining Applications	NIAs	Swarm Robotics	Other Biomimicry
Energy Consumption	High energy usage in drilling, excavation, and transportation operations.	Optimization of speed, torque, and energy parameters [29,37,39,41]	None	Energy-efficient designs [110,111,112,121,122,123,124,125]
Navigation and Mapping	Difficulty in navigating complex underground networks and generating real-time maps.	Real-time mapping optimization [43,44,45,46,47]	Drones for mapping [76,77,78,79,83,84,93,94,95,96]	None
Transport and Logistics	Inefficient ore transportation and equipment movement in large-scale operations.	Route planning [38,45,46,47]	Autonomous trucks (Automation) [67,68,69,70,71,72,73,74,89,90,91]	Adaptive modular designs [130,131,132,133,134,135,136,137]
Safety and Hazard Management	Risk of rockfalls, gas leaks, and other hazards requiring early detection.	None	Distributed monitoring [99]	Sensors for hazard detection [119,144,145]
Environmental Impact	Significant disturbance to ecosystems and soil due to mining activities.	None	Cooperative ecological restoration (autonomous revegetation, soil stabilization, distributed monitoring)	Burrowing technique [115,116,140,141,142]
Adaptability in Dynamic Environments	There are frequent changes in geology, ore quality, and equipment functionality.	Dynamic adjustments [48,50]	Task redistribution [93,94,95,96]	None
Scalability and Cost-Effectiveness	High costs and complexity in deploying advanced technologies at scale.	Efficient resource allocation [38,39,45,46,47]	Scalable systems	Cost-effective modular designs [117,128,129]

**Table 5:** Integrated Solutions to Mining Challenges, with Specific Contributions.

Mining Challenges	NIAs	Swarm Robotics	Other Biomimicry	Integration
Energy Consumption	✔		✔	Clam-inspired designs reduce excavation energy needs; PSO dynamically adjusts drilling parameters for energy efficiency.
Navigation and Mapping	✔	✔		Emesent drones and UX-1 robots use SLAM for autonomous mapping; ACO refines multi-robot data for accurate, real-time updates.
Transport and Logistics	✔	✔	✔	AHS and AutoMine ensure fleet coordination; PSO optimizes routes; mole-inspired designs enhance mobility in challenging terrains.
Safety and Hazard Management		✔	✔	Whisker-inspired sensors detect hazards; swarm robotics distributes risk monitoring.
Environmental Impact		✔	✔	Mole- and earthworm-inspired tunneling technologies minimize environmental impacts; NEXGEN SIMS robots assist in targeted environmental remediation.
Adaptability in Dynamic Environments	✔	✔	✔	ACO and GWO allow robots like UX-1 to adjust to unexpected changes, ensuring operational continuity while optimizing coordination and ensuring efficient mapping and hazard detection in flooded environments.
Scalability and Cost-Effectiveness	✔		✔	Modular designs like CRABOT reduce costs; PSO optimizes resource allocation for large-scale mining, ensuring scalability and economic feasibility.
